# CYP2J2 and EETs protect against pulmonary arterial hypertension with lung ischemia–reperfusion injury in vivo and in vitro

**DOI:** 10.1186/s12931-021-01891-w

**Published:** 2021-11-13

**Authors:** Yun Ding, Pengjie Tu, Yiyong Chen, Yangyun Huang, Xiaojie Pan, Wenshu Chen

**Affiliations:** Department of Thoracic Surgery, Fujian Provincial Hospital, Shengli Clinical Medical College of Fujian Medical University, Fuzhou, 350001 Fujian China

**Keywords:** CYP2J2, EETs, Pulmonary arterial hypertension, Lung ischemia–reperfusion injury

## Abstract

**Background:**

Cytochrome P450 epoxygenase 2J2 (CYP2J2) metabolizes arachidonic acid to epoxyeicosatrienoic acids (EETs), which exert anti-inflammatory, anti-apoptotic, pro-proliferative, and antioxidant effects on the cardiovascular system. However, the role of CYP2J2 and EETs in pulmonary arterial hypertension (PAH) with lung ischemia–reperfusion injury (LIRI) remains unclear. In the present study, we investigated the effects of CYP2J2 overexpression and exogenous EETs on PAH with LIRI in vitro and in vivo.

**Methods:**

CYP2J2 gene was transfected into rat lung tissue by recombinant adeno-associated virus (rAAV) to increase the levels of EETs in serum and lung tissue. A rat model of PAH with LIRI was constructed by intraperitoneal injection of monocrotaline (50 mg/kg) for 4 weeks, followed by clamping of the left pulmonary hilum for 1 h and reperfusion for 2 h. In addition, we established a cellular model of human pulmonary artery endothelial cells (HPAECs) with TNF-α combined with anoxia/reoxygenation (anoxia for 8 h and reoxygenation for 16 h) to determine the effect and mechanism of exogenous EETs.

**Results:**

CYP2J2 overexpression significantly reduced the inflammatory response, oxidative stress and apoptosis associated with lung injury in PAH with LIRI. In addition, exogenous EETs suppressed inflammatory response and reduced intracellular reactive oxygen species (ROS) production and inhibited apoptosis in a tumor necrosis factor alpha (TNF-α) combined hypoxia-reoxygenation model of HPAECs. Our further studies revealed that the anti-inflammatory effects of CYP2J2 overexpression and EETs might be mediated by the activation of PPARγ; the anti-apoptotic effects might be mediated by the PI3K/AKT pathway.

**Conclusions:**

CYP2J2 overexpression and EETs protect against PAH with LIRI via anti-inflammation, anti-oxidative stress and anti-apoptosis, suggesting that increased levels of EETs may be a promising strategy for the prevention and treatment of PAH with LIRI.

**Graphical Abstract:**

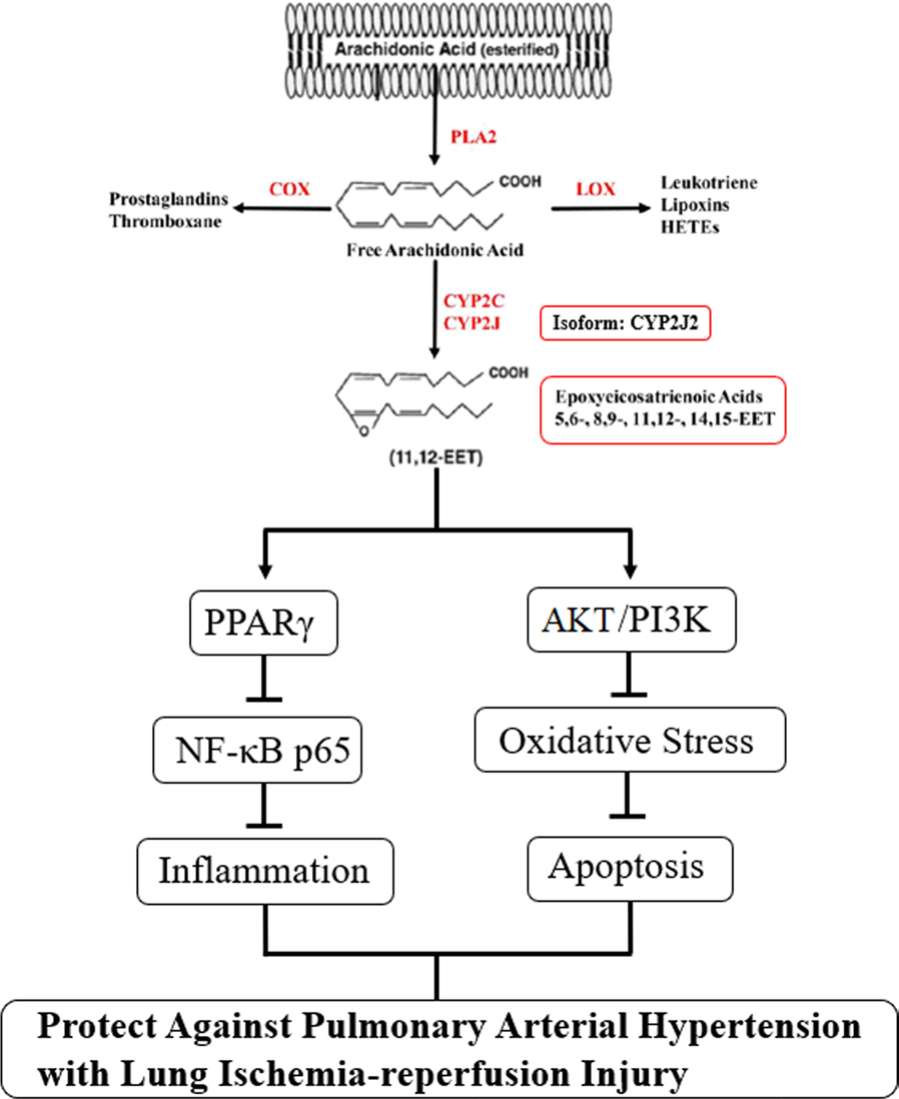

## Background

Lung ischemia–reperfusion injury (LIRI) is a frequent complication during cardiopulmonary surgery, such as lung transplantation, pulmonary artery reconstruction and extracorporeal circulation [1, 2]. The incidence of pulmonary arterial hypertension (PAH) secondary to cardiopulmonary disease is increasing each year with the aging of population [3], and thus many patients undergoing cardiothoracic surgery may have PAH. For these patients, how to reduce periprocedural LIRI and postoperative complications has become an important issue. However, there is no consensus protocol for reducing LIRI during lung ischemia–reperfusion in patients with PAH, and few studies have been performed on the effect of PAH with LIRI in animal models. Therefore, there is an urgent need for in-depth studies on LIRI in combination with PAH to understand the impact of PAH with LIRI comprehensively and to explore prevention and treatment strategies for PAH with LIRI.

The development of LIRI is a complex pathophysiological process involving inflammation, oxidative stress, intracellular calcium overload, apoptosis, and upregulation of cell surface membrane molecules [2, 4]. During these procedures, vascular endothelial cells are important mediators of LIRI, being not only the primary target cells of injury but also active effector cells. During ischemia, the synthesis and secretion of pro-inflammatory mediators and reactive oxygen species (ROS) by vascular endothelial cells increase, while nitric oxide synthase (NOS) and prostacyclin synthesis decrease, resulting in endothelial dysfunction [4, 5]. After reperfusion, pro-inflammatory mediators and oxygen free radicals can activate neutrophils, platelets and nitric oxide (NO) signalling pathways, which together act on vascular endothelial cells and cause vascular damage, followed by increased pulmonary vascular permeability, microcirculatory resistance and pulmonary oedema [6, 7]. Therefore, improving vascular endothelial cell function has become an important target for the prevention and treatment of LIRI.

Vascular endothelial dysfunction also plays a central role in the pathogenesis of PAH, which leads to structural and functional abnormalities in the pulmonary vasculature through a series of cascading reactions that promote vasoconstriction, smooth muscle proliferation and inflammation [3]. Long-term constriction of small pulmonary arteries causes hypertrophy and proliferation of smooth muscle and fibroblasts in the pulmonary vascular wall, resulting in narrowing of the lumen and increased vascular resistance [8]. Besides, collagen and elastic fibres are deposited in the pulmonary vascular wall, making the vessels less elastic and reducing diastolic function [9], increasing small pulmonary vascular resistance, slowing down blood flow during reperfusion, affecting the effect of reperfusion and aggravating lung injury. In addition, PAH can cause thrombosis [10], which exacerbates intrapulmonary thrombosis during lung-ischemia and affects reperfusion. Therefore, it is equally important to improve PAH-related endothelial cell injury when preventing and treating PAH with LIRI.

Cytochrome P450 epoxygenase (CYP) 2J2 is highly expressed in the heart and lungs [11]. CYP2J2 metabolizes free arachidonic acid in vivo to four isomeric epoxyeicosatrienoic acids (EETs). The CYP2J2-EETs axis plays an important role in maintaining the vascular endothelial cell barrier and vascular function [12, 13]. Our previous studies have shown that CYP2J2 and its metabolites, EETs, could protect against LIRI via inhibiting pulmonary artery endothelial cell inflammation, oxidative stress and apoptosis, consequently reducing parenchymal inflammatory cell infiltration and improving lung function [14, 15]. In addition, CYP2J2 overexpression can reduce monocrotaline induced PAH in rats [16] and inhibit TNF-α-induced apoptosis of pulmonary artery endothelial cells and TGF-β1-induced proliferation and migration of pulmonary artery smooth muscle cells [17]. Based on the above studies, CYP2J2 overexpression and its metabolites, EETs, attenuated both PAH and LIRI, but the effect on LIRI in combination with PAH is unclear. Therefore, the aim of our study was to determine whether CYP2J2 and EETs exerted protective effects against LIRI affected by PAH and to elucidate their mechanism of action through in vivo and in vitro experiments.

## Methods

### Construction of gene delivery vectors

Recombinant adeno-associated virus (rAAV) carrying CYP2J2 gene or green fluorescent protein (GFP) was constructed by Fuzhou Zolgene Co., Ltd. and prepared for use after titer determination.

### Experimental animal grouping

Thirty-five clean-grade 8-week-old male SD rats, weighing 250–300 g, were purchased from the Experimental Animal Center of Fujian Medical University. Rats were randomly divided into 7 groups (5 rats per group): Control group, PAH group, PAH + Sham group, PAH + IR group, rAAV-GFP gene-transfected PAH + IR group (PAH + IR + GFP), rAAV-CYP2J2 gene-transfected PAH + IR group (PAH + IR + 2J2) and rAAV-CYP2J2 gene transfected + C26 of PAH + IR group (PAH + IR + 2J2 + C26).

### Animal model interventions

Except for the Control group, all rats were given a single intraperitoneal injection of monocrotaline (MCT, Sigma) at 50 mg/kg. The Control group was given a single intraperitoneal injection of the corresponding dose of saline.

24 h after MCT injection, rats in the PAH + IR + GFP, PAH + IR + 2J2 and PAH + IR + 2J2 + C26 groups received tail vein injection of rAAV carrying the corresponding gene at 1*10^7^ PFU/kg; the remaining groups received tail vein injection of the related volume of saline. Meanwhile, rats in the PAH + IR + 2J2 + C26 group started C26, a selective CYP2J2 inhibitor [18], gavage at a dose of 1.5 mg/kg/day until the ischemia–reperfusion (IR) operation.

**Fig. 1:**
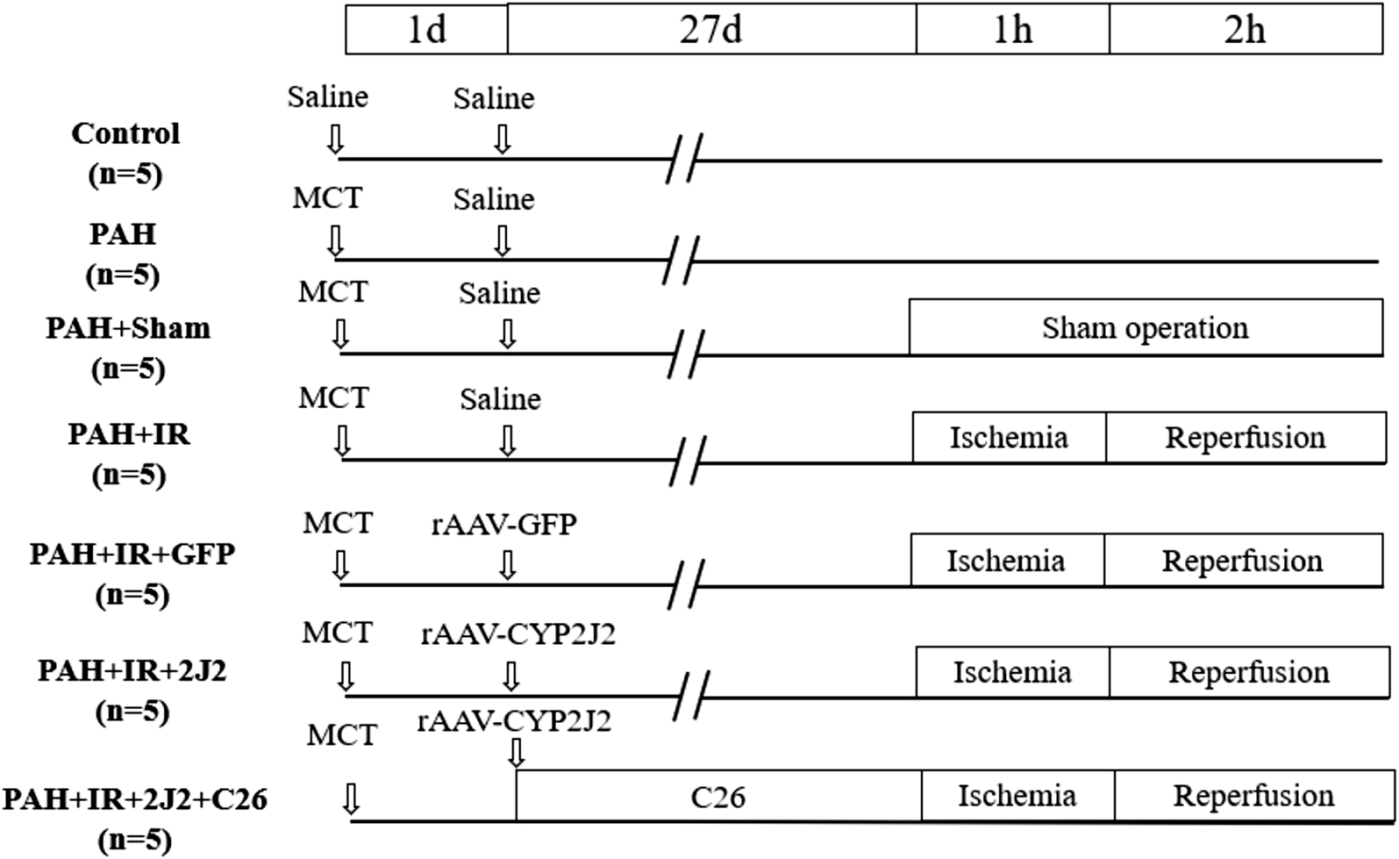
Schematic diagram of animal grouping and interventions

Four weeks after MCT injection, IR was performed in PAH + IR, PAH + IR + GFP, PAH + IR + 2J2, and PAH + IR + 2J2 + C26 groups. The rats were anesthetized by intraperitoneal injection of urethane solution (1 g/kg). The left pulmonary hilum (including the left main bronchus, pulmonary artery and pulmonary vein) was clamped with a noninvasive arterial clip, resulting in complete ischemia and hypoxia of the left lung for 1 h. Afterwards, the vascular clamp was released to restore the ventilation and perfusion of the left lung for 2 h. PAH + Sham group underwent the same thoracotomy procedure and hilar dissection but without hilar block. Thoracotomy procedure was not performed in control and PAH groups. A schematic diagram of grouping and interventions is shown in Fig. 1.

### Hemodynamic measurements

The mean pulmonary artery pressure (mPAP) and right ventricular systolic pressure (RVSP) were measured after 2 h of reperfusion. The heparinized indwelling needle was connected to the biosignal acquisition system via a pressure transducer. The indwelling needle was inserted into the right ventricle via the right ventricular apex. The needle core was withdrawn after 2.5 mm of insertion, and the indwelling needle was continued to be pushed inward by 2.5–5 mm to the right ventricle and pulmonary artery to record RVSP and mPAP. Rats were euthanized by exsanguination via the vena cava immediately after measurement.

### Pathological analysis

After the rats were euthanized, the left lungs were removed. A portion of the left lung tissue was cut and fixed with paraformaldehyde at room temperature for 24 h and then embedded in paraffin. The lung tissues were cut horizontally into 5 μm thick sections and stained with hematoxylin–eosin (HE). The morphology of pulmonary vessels and the extent of lung tissue injury were observed using a high-powered microscope. In each high magnification (× 200) field, the lung injury was scored as follows [19]: 0, no apparent injury; 1, mild injury; 2, moderate injury; 3, severe injury, based on the extent of pulmonary hemorrhage, pulmonary edema and interstitial inflammatory cell infiltration, and the three scores were summed to obtain the lung injury score for this visual field. Three sections of each lung tissue were made and three high magnification (× 200) fields of view were observed in each area, and the average value was taken as the lung injury score of the rat.

### TUNEL staining

Apoptotic cells in lung tissue were detected in situ by TUNEL staining with a kit (Roche, Germany). Under the microscope, cells with brown nuclei stained positive for TUNEL staining were considered apoptotic cells. The results were counted by two researchers in a double-blind manner under at least five high-magnification fields (× 400). The number of apoptotic cells and their proportion to the total number of cells in each area were calculated.

### Wet weight/dry weight (W/D)

A portion of the left lung tissue was cut for W/D determination. The wet weight (W) was measured by absorbing the liquid from the surface of the tissue with filter paper, and the dry weight (D) was measured after drying in an oven at 65 ℃ for 72 h, and the W/D value was calculated.

### Cell culture

Human pulmonary artery endothelial cells (HPAECs, ScienCell, USA) were cultured in endothelial cell-specific culture medium containing 5% fetal bovine serum, 1% endothelial cell growth factor and 1% penicillin/streptomycin. Cell passaging was performed once the confluency reached approximately 90%.

### Simulation of PAH with LIRI model in vitro and cell processing

Cells were transferred to 6-well plates after passaging and incubated in a 37 °C normoxic incubator (5% CO_2_, 95% air, 95% humidity). When the monolayer cells grew to 80% confluence, 50 ng/ml TNF-α was added to simulate the endothelial cell injury model. 4 h later, the medium was removed and the cells were washed twice with phosphate buffer (PBS). Then the cells were pretreated with 14,15-EEZE (10 μmol/l, Cayman Chemicals), a selective EET antagonist [20], for 1 h. After that, 14,15-EETs (1 μmol/l, Sigma) or lysis medium (DMSO) were added to the culture medium. 1 h later, anoxia/reoxygenation (AR) was performed. The cell culture medium was replaced with a sugar-free serum-free medium and incubated in a 37 °C anoxic incubator (5% CO_2_, 95% nitrogen). 8 h later, the cells were replaced with a complete cell culture medium and incubated in a normoxic incubator for 16 h. When the cell growth reached 80% confluence in the normoxic group, the corresponding reagents were added and incubation was continued in the normoxic incubator for 24 h without AR.

### Cell viability assay

Cell viability was detected by the CCK8 kit (Beyotime, China), and the effect of different intervention methods on cell viability was observed in each group. The impact of EETs on the cell viability level of TNF-α combined with AR on endothelial cells was assessed by measuring the absorbance of 450 nm with a microplate reader (Bio-Tek ELX800, USA).

### ROS measurement

The intracellular ROS level after AR was detected using DCFH-DA fluorescent probe (Sigma-Aldrich, USA). After the cell intervention, the cells were incubated with 10 μmol/l DCFH-DA for 20 min at 37 °C. Immediately after staining, the cells were collected and the fluorescence intensity of DCF was measured by flow cytometry.

### Flow cytometry detection of apoptosis

After the cell intervention, the cells were collected and labeled with Annexin V-FITC/PI kit (NanJing KeyGen Biotech Co., Ltd., China) and then detected by flow cytometry for apoptosis.

### Mitochondrial membrane potential detection

The mitochondrial membrane potential assay kit (Beyotime, China) was used to analyze the extent of mitochondrial membrane damage by flow cytometry using a mitochondrial membrane potential assay probe (JC-1) for labeling.

### ELISA assay

The levels of inflammatory factors IL-1β, IL-6, IL-10 and TNF-α in rat serum and cell supernatant were measured by ELISA kits (Shanghai Lianshuo Biological Technology Co. Ltd., China), which were operated according to the ELISA kit instructions.

### Western blot assay

Western blots were performed to detecte the cytoplasmic proteins extracted from rat lung tissues and cells. The primary antibodies used were: CYP2J2 (Biogot Technology Co., Ltd., China); PPARγ, phosphorylated NF-κB p65 (p-NF-κB p65, Ser536), total NF-κB p65 (t-NF-κB p65), phosphorylated AKT (p-AKT, Ser473), total AKT(t-AKT) and PI3K (Shanghai Lianshuo Biological Technology Co. Ltd., China).

### Statistical analysis

SPSS 21.0 software (SPSS, Chicago, USA) was applied for statistical analysis and GraphPad Prism 8.0.1 software (GraphPad Software, CA, USA) was used for plotting. The measurement data were expressed as mean ± standard deviation. One-way ANOVA with Bonferroni post hoc test was used for comparison between multiple groups, and *P* < 0.05 was considered a statistically significant difference.

## Results

### CYP2J2 gene transfection increases the content of CYP2J2 protein and plasma EETs in rat lung tissue

**Fig. 2:**
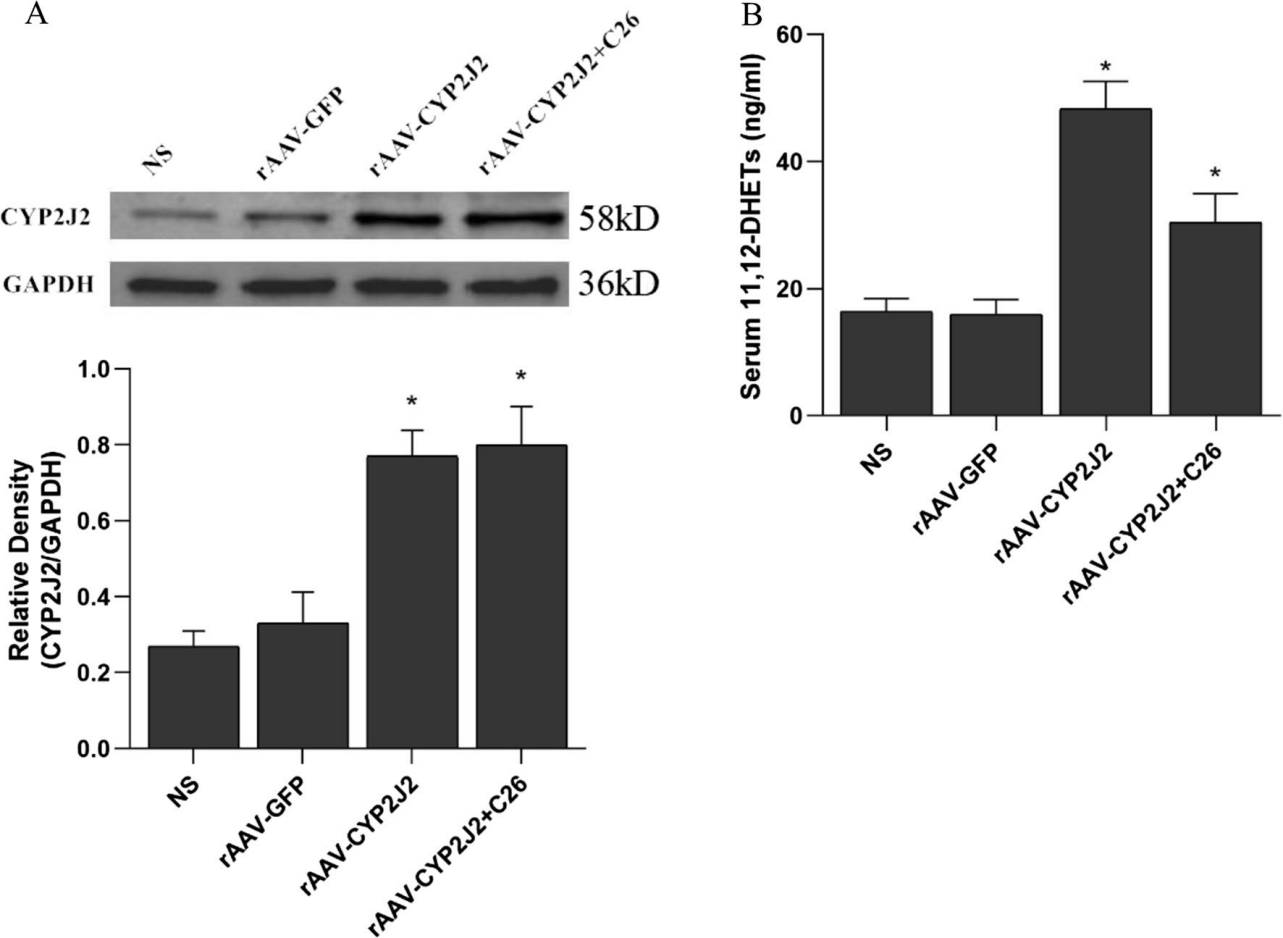
Effect of CYP2J2 gene transfection on the content of CYP2J2 protein in rat lung tissues and EETs in plasma. **A** The upper graph shows the representative Western blot images of CYP2J2 protein in lung tissues of PAH with LIRI rats after administration of different interventions in 4 groups by Western blot, and the lower graph shows the relative grayscale values of CYP2J2 protein in lung tissues of each group (n = 3; *P < 0.05 vs. NS or rAAV-GFP). **B** Levels of plasma 11,12-DHETs in each group (n = 3; *P < 0.05 vs. NS or rAAV-GFP). *NS* normal saline

The expression of CYP2J2 protein in rat lung tissue was detected by Western blot 4 weeks after gene transfection (Fig. 2A). In addition, CYP2J2 metabolizes arachidonic acid to EETs, which are unstable in vivo and easily oxidized to dihydroxyeicosatrienoic acids (DHETs), so the level of 11,12-DHETs in rat plasma was tested to indirectly reflect the level of EETs (Fig. 2B). The results showed that lung tissue CYP2J2 protein and plasma 11,12-DHETs levels were significantly higher in rats injected with rAAV-CYP2J2 than in non-rAAV-CYP2J2-injected rats with PAH with LIRI.

### CYP2J2 overexpression decreased mPAP and RVSP in rats

**Fig. 3:**
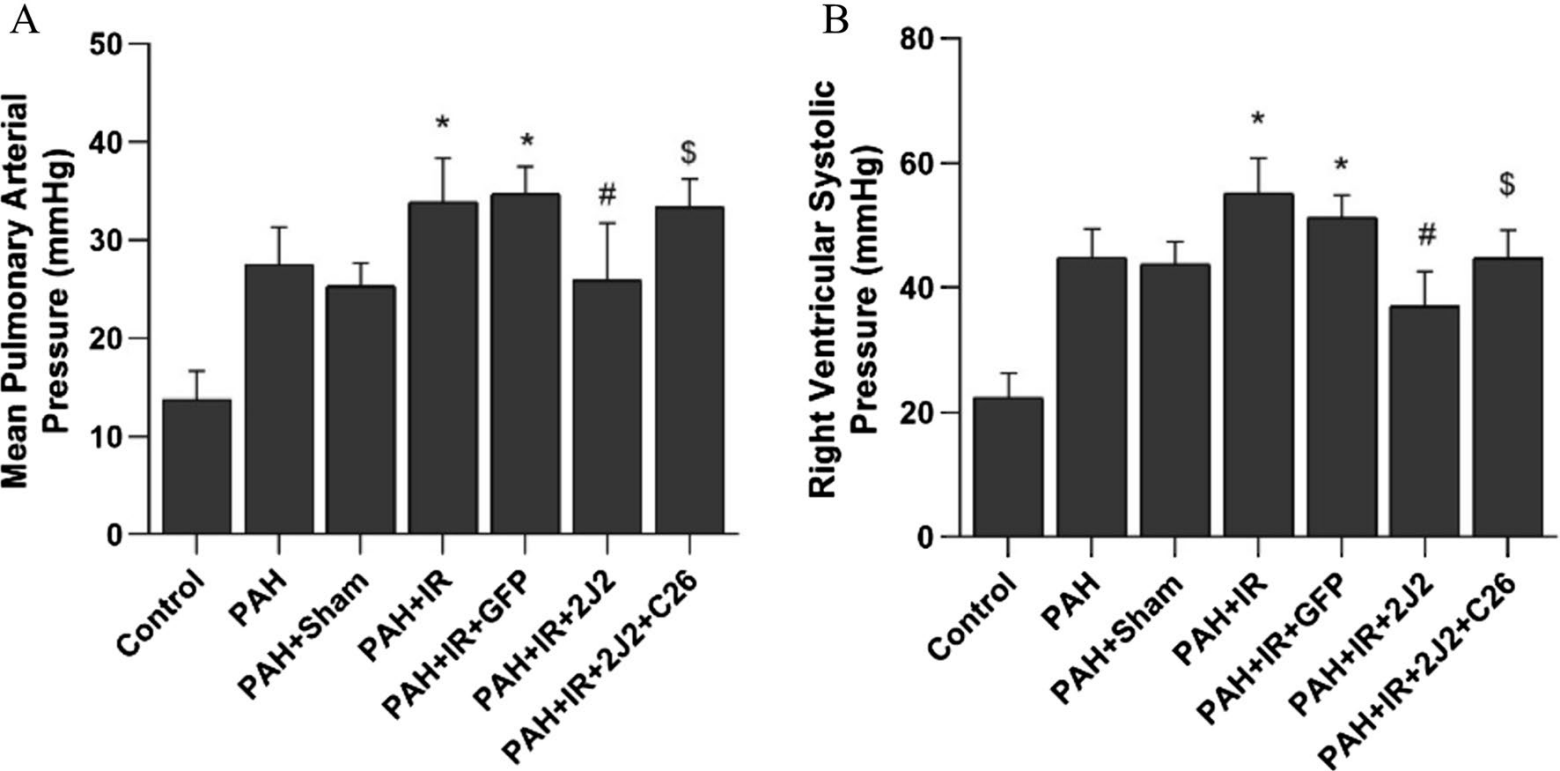
Comparison of mean pulmonary artery pressure (**A**) and right ventricular systolic pressure (**B**) in each group (n = 5; *P < 0.05 vs. Control, PAH or PAH + Sham group, ^#^*P* < 0.05 vs. PAH + IR or PAH + IR + GFP group)

As shown in Fig. 3A and B, mPAP and RVSP were detected after IR operation. We found that MCT injection combined with IR operation significantly increased mPAP and RVSP in rats, and CYP2J2 overexpression decreased mPAP and RVSP in rats with PAH with LIRI model, but this effect was inhibited by C26 (the selective inhibitor of CYP2J2).

### CYP2J2 overexpression attenuated the changes in histopathology

**Fig. 4:**
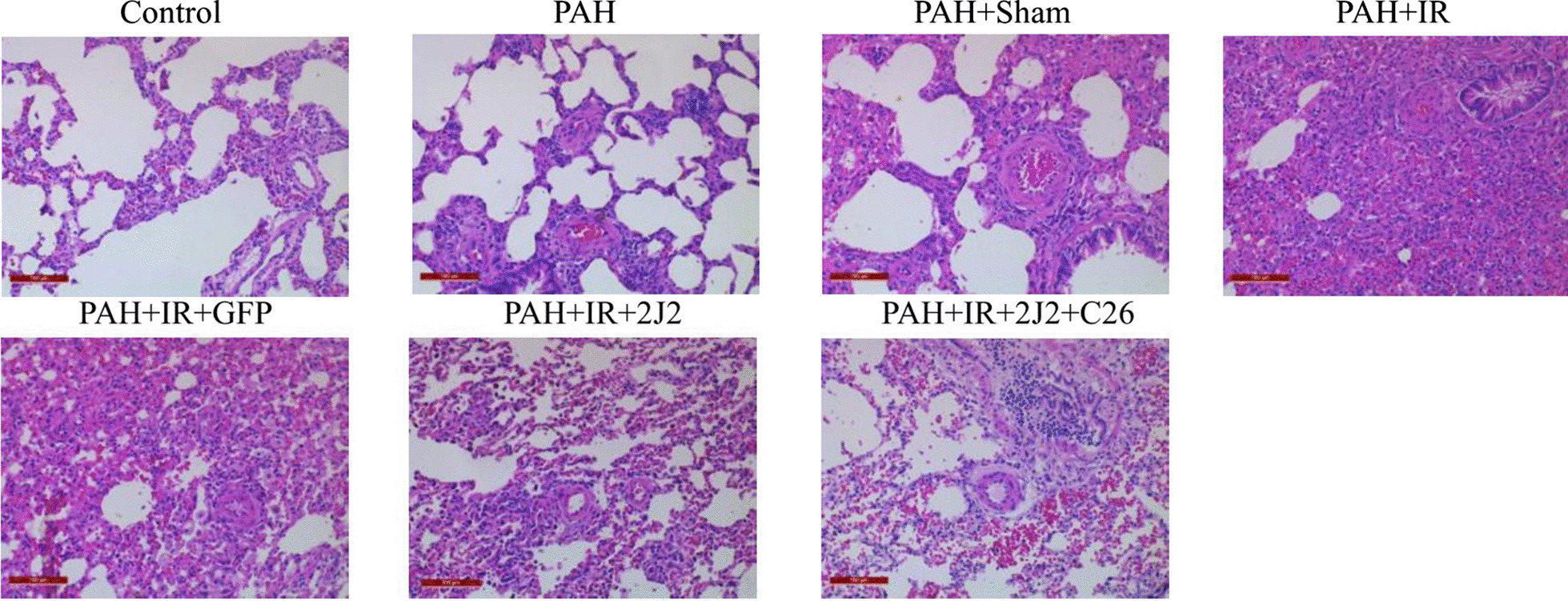
HE staining of the left lungs in each group (original magnification, × 200). Scale bar = 100 μm

As shown in Fig. 4, under light microscopy (× 200), sections of specimens from each group processed by MCT showed varying degrees of vascular endothelial layer hyperplasia, thickening of the smooth muscle layer and elastic fiber layer of small pulmonary artery vessels, and reduction of the vascular lumen, suggesting pulmonary vascular remodeling. No significant inflammatory cell infiltration between lung tissues or around small vessels was observed in Control group. In contrast, inflammatory cell infiltration around small vessels and in the alveolar space and interstitium, intra-alveolar hemorrhage, and significant widening of the alveolar septum were detected in PAH + LIRI group. Furthermore, these changes were significantly attenuated by CYP2J2 overexpression, while C26 inhibited the effect of CYP2J2.

### CYP2J2 overexpression attenuates lung injury in vivo

**Fig. 5:**
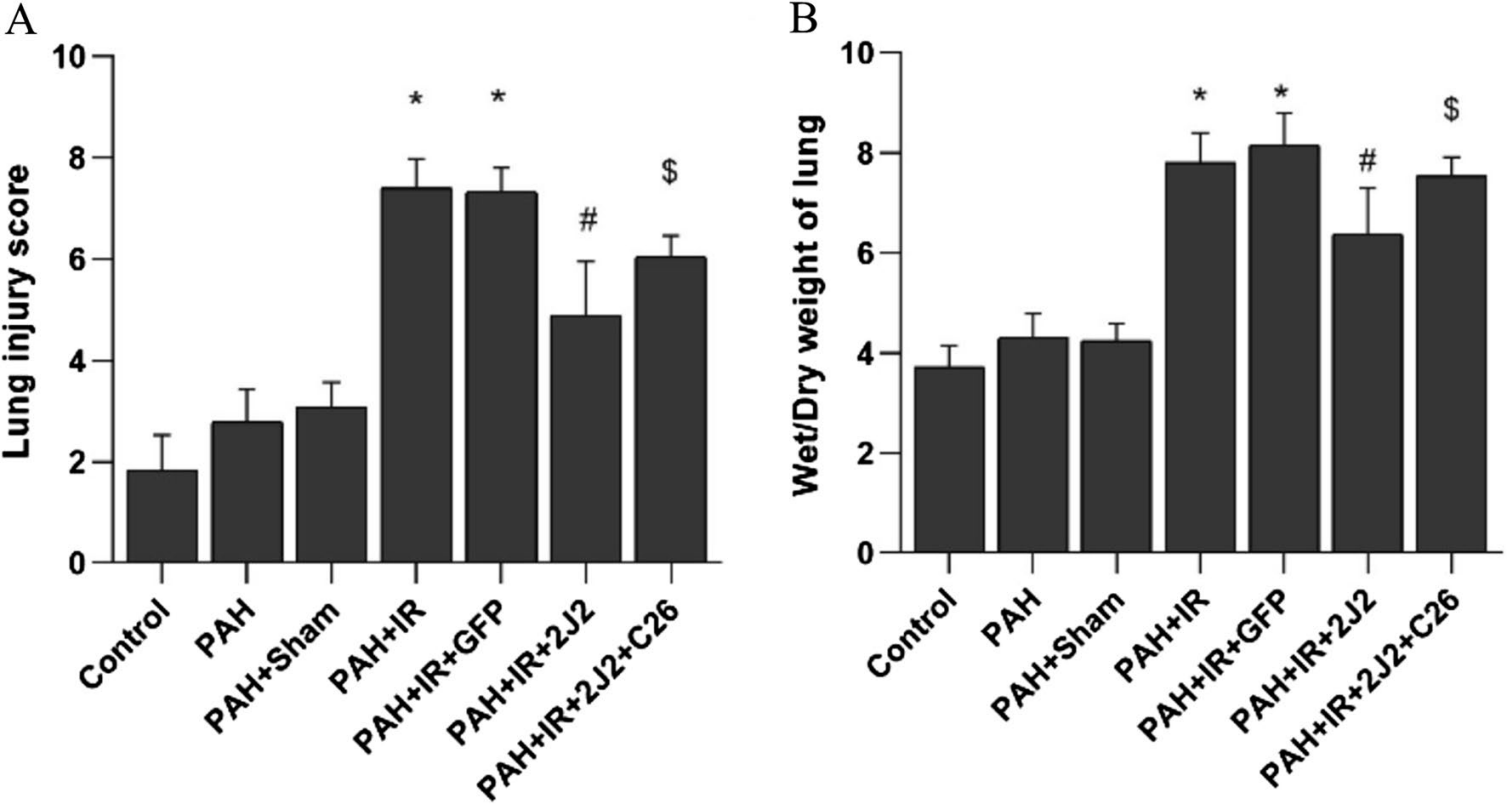
Comparison of left lung injury scores (**A**) and wet-to-dry weight ratios (**B**) in each group (n = 5; *P < 0.05 vs. Control, PAH or PAH + Sham group, ^#^*P* < 0.05 vs. PAH + IR or PAH + IR + GFP group, ^$^*P* < 0.05 vs. PAH + IR + 2J2 group)

Elevated W/D is associated with inflammation and edema and can effectively reflect lung injury. Therefore, we assessed lung injury by lung injury score and W/D. As shown in Fig. 5A and B, IR operation significantly increased the lung injury score and W/D ratio, while CYP2J2 transfection attenuated this change, which was inhibited by C26.

### CYP2J2 overexpression inhibits apoptosis in vivo

**Fig. 6:**
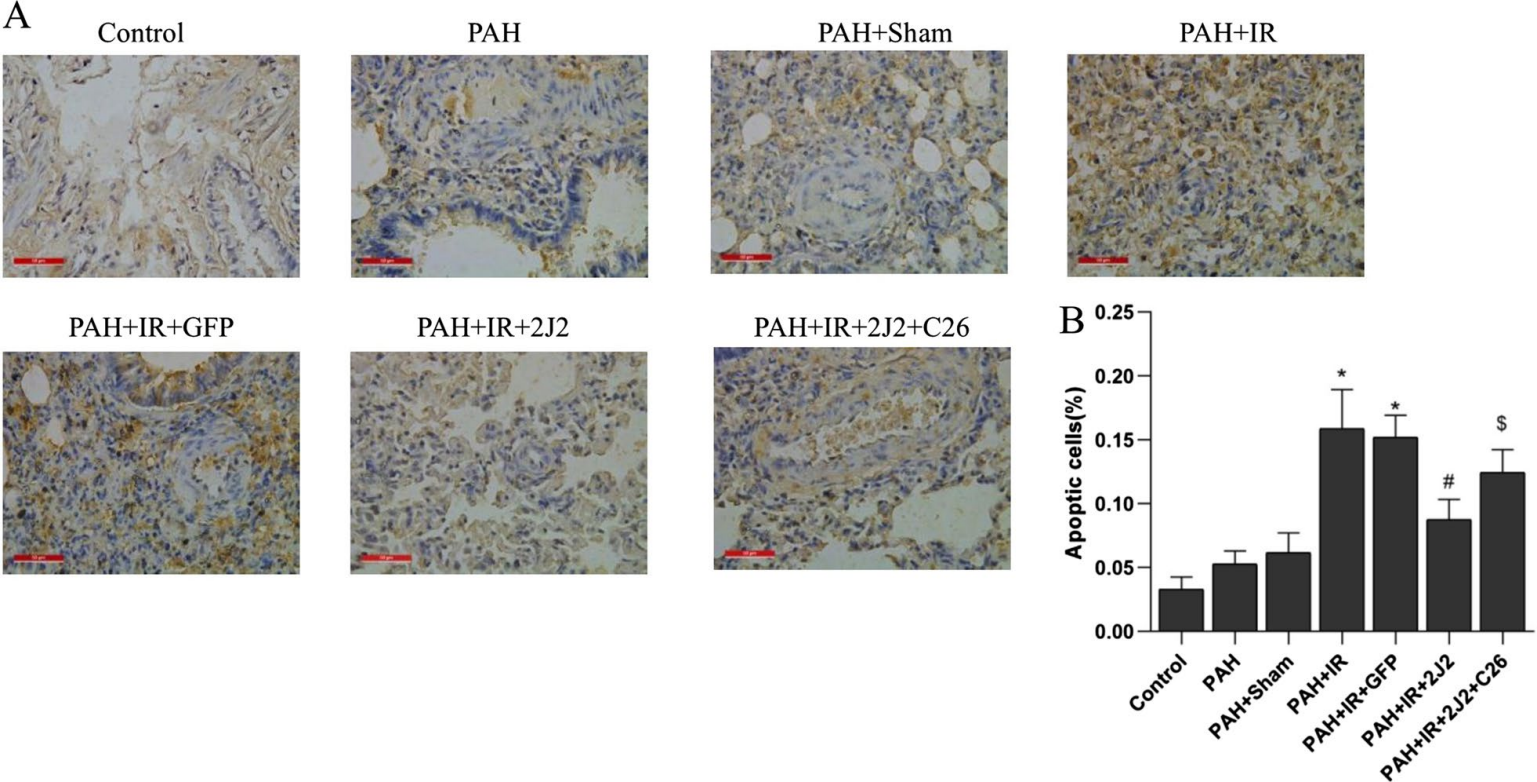
**A** Apoptotic cells in the left lungs were identified through TUNEL staining (original magnification, × 400). Scale bar = 50 μm. **B** The mean percentage of TUNEL-positive cells in each group. (n = 5; *P < 0.05 vs. Control, PAH or PAH + Sham group, ^#^*P* < 0.05 vs. PAH + IR or PAH + IR + GFP group, ^$^*P* < 0.05 vs. PAH + IR + 2J2 group)

As shown in Fig. 6A and B, the percentage of TUNEL-positive cells was significantly increased in the PAH + IR group compared with the Control and PAH + Sham groups, which was alleviated by CYP2J2 transfection. It suggested that CYP2J2 overexpression inhibited apoptosis in lung tissue of PAH with LIRI.

### EETs increase the viability of HPAECs treated with TNF-α combined with AR

**Fig. 7:**
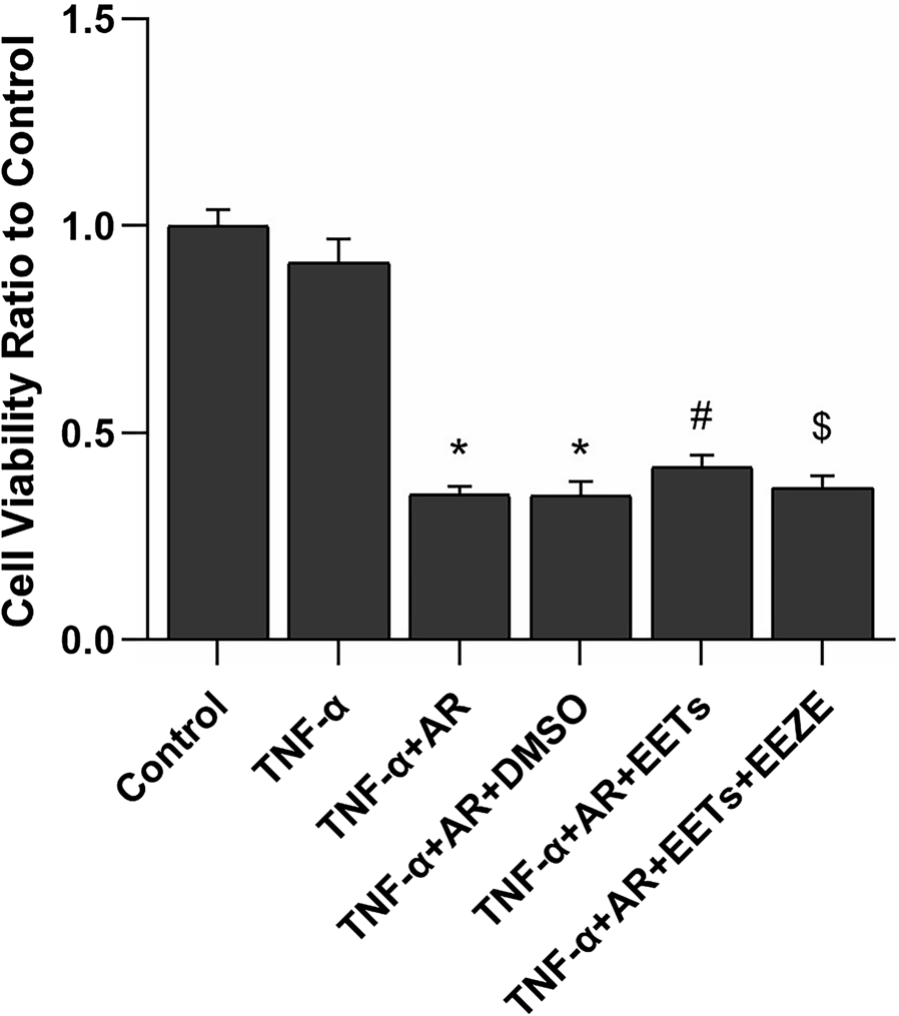
Effect of exogenous EETs on the viability of HPAECs after TNF-α combined with anoxia/reoxygenation. Cell viability was detected by CCK-8 kit (n = 5; *P < 0.05 vs. Control or TNF-α group, ^#^*P* < 0.05 vs. TNF-α + AR or TNF-α + AR + DMSO group, ^$^*P* < 0.05 vs. TNF-α + AR + EETs group)

As shown in Fig. 7, CCK8 kit was used to assess the effect of TNF-α combined with AR (anoxia for 8 h and reoxygenation for 16 h) on cell viability and the role of exogenous EETs. The results showed that TNF-α combined with AR of HPAECs significantly decreased cell viability, which could be prevented by EETs.

### EETs inhibit ROS production in HPAEC treated with TNF-α combined with AR

**Fig. 8:**
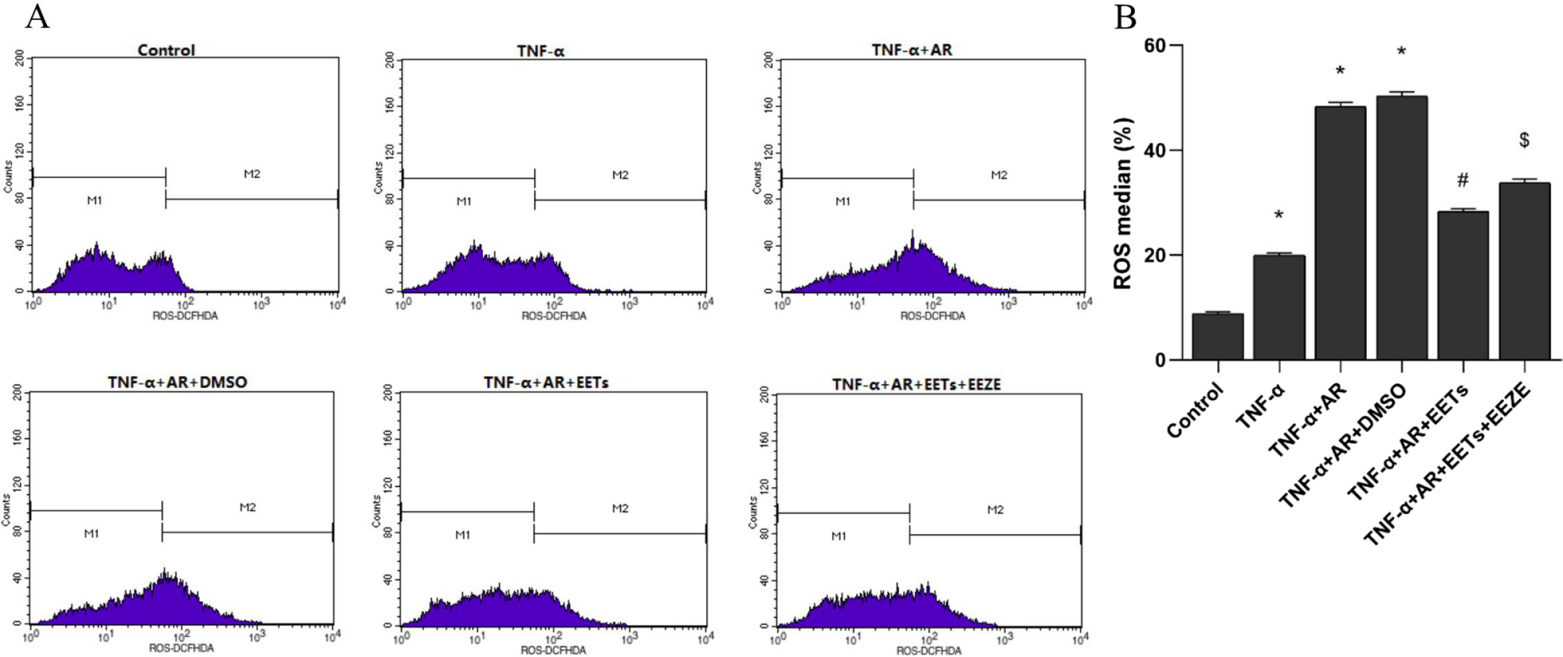
Exogenous EETs inhibit ROS production induced by TNF-α combined with anoxia/reoxygenation in HPAECs. **A** ROS were detected by flow cytometry, and the M2 region represents ROS-positive cells. **B** Plots represent the mean percentage of ROS-positive cells in each group (n = 3; *P < 0.05 vs. Control group, ^#^*P* < 0.05 vs. TNF-α + AR or TNF-α + AR + DMSO group, ^$^*P* < 0.05 vs. TNF-α + AR + EETs group)

The mean fluorescence intensity of DCFH-DA detected by flow cytometry represented the ROS level. The results showed that TNF-α increased ROS production in HPAECs, and AR further increased ROS production in TNF-α-treated HPAECs. In contrast, EETs significantly attenuated the TNF-α + AR-induced increase in ROS levels (Fig. 8A and B). However, the effect of EETs was inhibited when cells were pretreated with 14,15-EEZE (the selective inhibitor of EETs).

### EETs inhibit apoptosis and mitochondrial transmembrane potential of HPAECs treated with TNF-α combined with AR

**Fig. 9:**
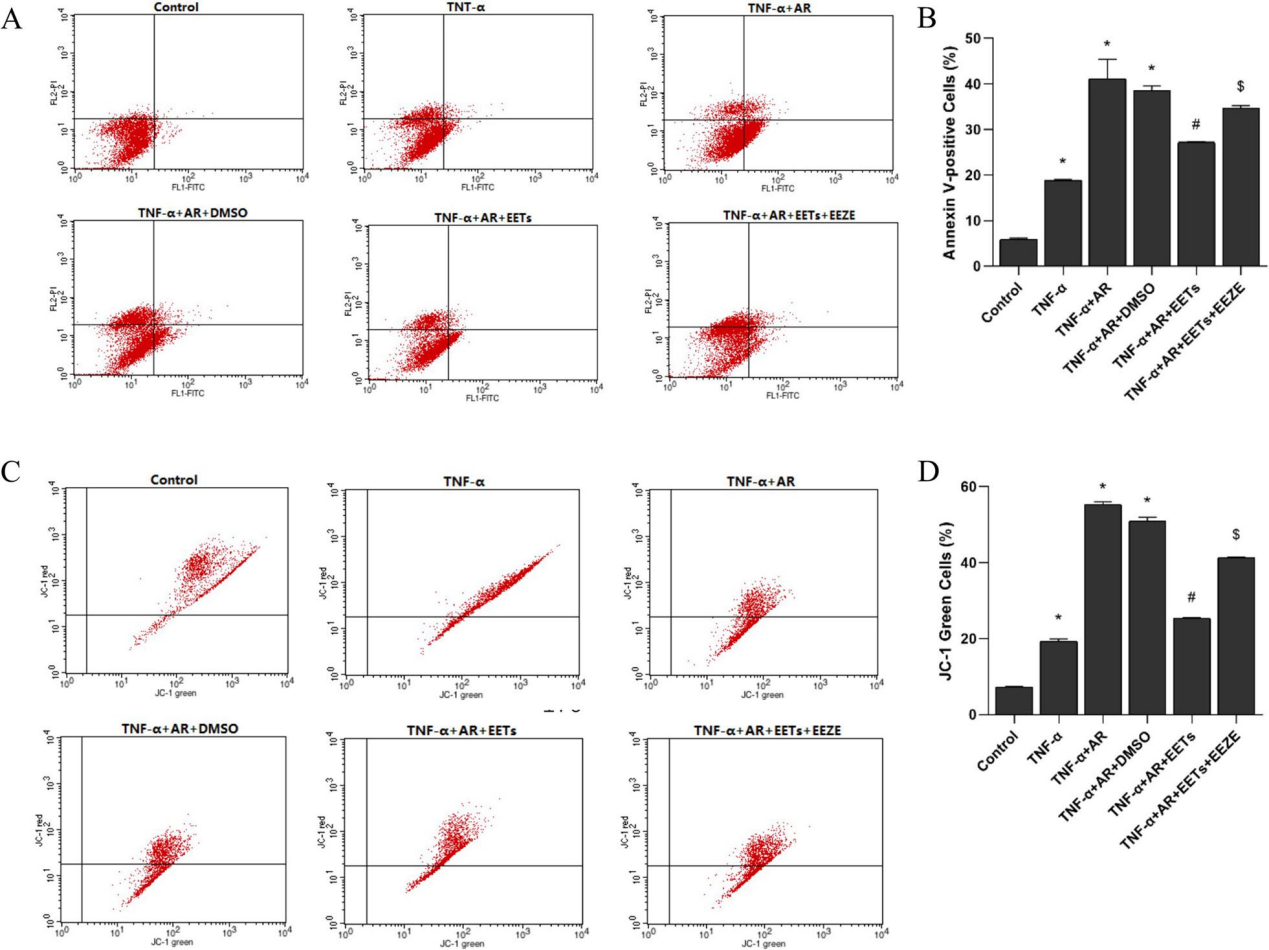
Exogenous EETs inhibit TNF-α combined with anoxia/reoxygenation-induced apoptosis and mitochondrial damage in HPAECs. **A** Annexin V-positive HPAECs stained by Annexin V-FITC and PI were counted by flow cytometry. Lower right quadrant: number of Annexin V-positive cells for early apoptosis; upper right quadrant: number of Annexin V-positive cells for late apoptosis or necrosis; upper left quadrant: number of PI-positive cells for late apoptosis or necrosis; lower left quadrant: number of PI-positive cells for early apoptosis. **B** The mean percentage of Annexin V-positive cells in each group (n = 3; *P < 0.05 vs. Control group, ^#^*P* < 0.05 vs. TNF-α + AR or TNF-α + AR + DMSO group, ^$^*P* < 0.05 vs. TNF-α + AR + EETs group). **C** The mitochondrial transmembrane potential was measured by JC-1 and analyzed by flow cytometry. Upper right quadrant: cells with normal mitochondrial function, which have diminished red fluorescence. Lower right quadrant: cells with loss of mitochondrial transmembrane potential with bright green fluorescence. **D** The mean percentage of cells with loss of mitochondrial transmembrane potential in each group (n = 3; *P < 0.05 vs. Control group, ^#^*P* < 0.05 vs. TNF-α + AR or TNF-α + AR + DMSO group, ^$^*P* < 0.05 vs. TNF-α + AR + EETs group)

The effect of exogenous EETs on the level of apoptosis in HPAECs was observed by flow cytometry using Annexin V-FITC/PI assay (Fig. 9A and B), and the alteration of mitochondrial transmembrane potential associated with apoptosis was assessed by flow cytometry (Fig. 9C and D). The results showed that exogenous EETs reduced TNF-α combined with AR-induced apoptosis in HPAECs, while EEZE inhibited the protective effect of EETs.

### CYP2J2 overexpression and exogenous EETs decrease the level of inflammatory factors in vivo and vitro

**Fig. 10:**
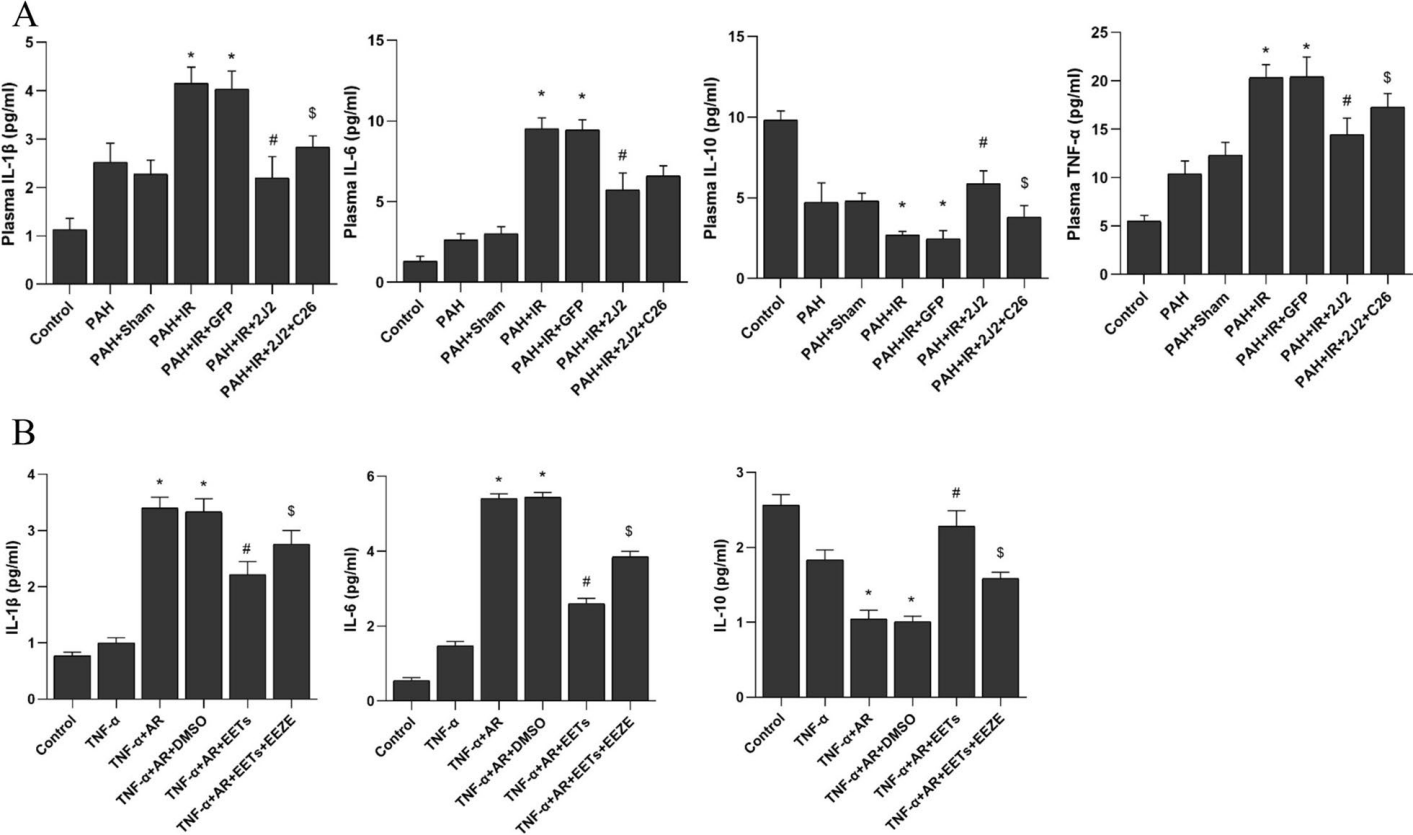
Effect of CYP2J2 overexpression and exogenous EETs on inflammatory factors. **A** Effect of CYP2J2 overexpression on plasma inflammatory factors in rats (n = 5; *P < 0.05 vs. Control, PAH or PAH + Sham group, ^#^*P* < 0.05 vs. PAH + IR or PAH + IR + GFP group, ^$^*P* < 0.05 vs. PAH + IR + 2J2 group). **B** Effect of exogenous EETs on inflammatory factors in the supernatant of HPAECs cells (n = 5; *P < 0.05 vs. Control or TNF-α group, ^#^*P* < 0.05 vs. TNF-α + AR or TNF-α + AR + DMSO group, ^$^*P* < 0.05 vs. TNF-α + AR + EETs group)

PAH complicated with IR was simulated in vivo and in vitro, CYP2J2 transfection and exogenous EETs intervention were respectively administered. As shown in Fig. 10, the levels of pro-inflammatory factors IL-1β and IL-6 were significantly increased and the level of anti-inflammatory factor IL-10 was decreased by both in vivo and in vitro experimental modeling manipulations. In addition, the level of TNF-α was significantly increased by in vivo experimental modeling manipulations. The changes of the above-mentioned inflammatory factors were significantly inhibited by CYP2J2 overexpression and exogenous EETs in vivo and in vitro.

### The anti-inflammatory effects of CYP2J2 overexpression and exogenous EETs are mediated by PPARγ activation

**Fig. 11:**
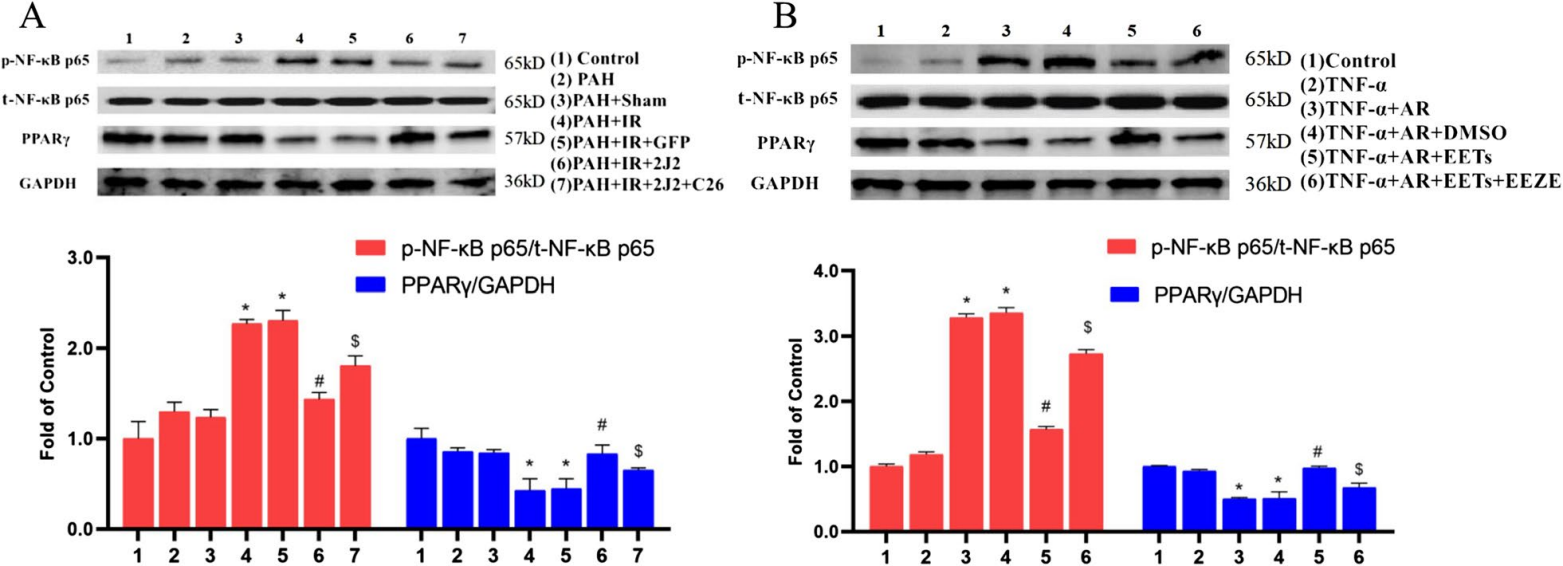
Effect of CYP2J2 overexpression and exogenous EETs on the expression of inflammation-related proteins. **A** Effect of CYP2J2 overexpression on inflammation-related proteins in rat lung tissues. The upper graph shows the representative Western blot plots of PPARγ, p-NF-κB p65 (Ser536), and t-NF-κB p65 in rat lung tissue of each group, and the lower graph shows the fold change of p-NF-κB p65/t-NF-κB p65 and PPARγ/GAPDH in each group to the relative density of Control group. Fold change was calculated by normalizing to control (fold change of control is 1). (n = 3; *P < 0.05 vs. Control, PAH or PAH + Sham group, ^#^*P* < 0.05 vs. PAH + IR or PAH + IR + GFP group, ^$^*P* < 0.05 vs. PAH + IR + 2J2 group). **B** Effect of exogenous EETs on inflammation-associated proteins in HPAECs. The upper graph shows the representative Western blot plots of PPARγ, p-NF-κB p65 (Ser536), and t-NF-κB p65 in each group of HPAECs, and the lower graph shows the fold change of p-NF-κB p65/t-NF-κB p65 and PPARγ/GAPDH in each group to the relative density of Control group. Fold change was calculated by normalizing to control (fold change of control is 1). (n = 3; *P < 0.05 vs. Control or TNF-α group, ^#^*P* < 0.05 vs. TNF-α + AR or TNF-α + AR + DMSO group, ^$^*P* < 0.05 vs. TNF-α + AR + EETs group)

We further examined PPARγ, p-NF-κB p65 and t-NF-κB p65. As shown in Fig. 11A and B, the levels of PPARγ were significantly decreased and the level of p-NF-κB p65 was increased by both in vivo and in vitro experimental modeling manipulations. The CYP2J2 overexpression and exogenous EETs significantly promoted the activation of PPARγ in vivo and in vitro.

### The anti-oxidative and anti-apoptotic effects of CYP2J2 overexpression and exogenous EETs are mediated by PI3K/AKT signaling pathway

**Fig. 12:**
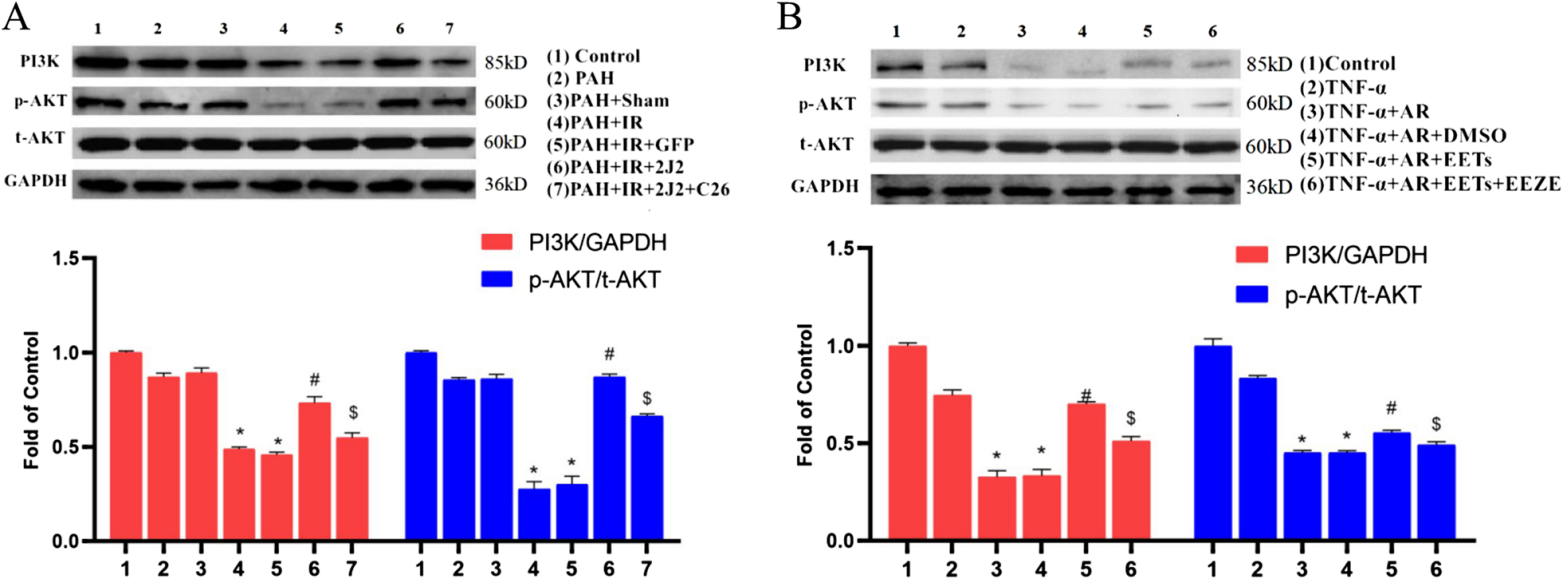
Effect of CYP2J2 overexpression and exogenous EETs on PI3K/AKT signaling pathway. **A** CYP2J2 overexpression significantly promoted the activation of PI3K/AKT signaling pathway in vivo. The upper graph shows the representative Western blot plots of PI3K, p-AKT (Ser473) and t-AKT in rat lung tissues, and the lower graph shows the fold change of PI3K/GAPDH and p-AKT/t-AKT in each group to the relative density of Control group. Fold change was calculated by normalizing to control (fold change of control is 1). (n = 3; *P < 0.05 vs. Control, PAH or PAH + Sham group, ^#^*P* < 0.05 vs. PAH + IR or PAH + IR + GFP group, ^$^*P* < 0.05 vs. PAH + IR + 2J2 group). **B** Exogenous EETs significantly promoted the activation of PI3K/AKT signaling pathway in vitro. The upper graph shows the representative Western blot plots of PI3K, p-AKT (Ser473) and t-AKT in HPAECs, and the lower graph shows the fold change of PI3K/GAPDH and p-AKT/t-AKT in each group to the relative density of Control group. Fold change was calculated by normalizing to control (fold change of control is 1). (n = 3; *P < 0.05 vs. Control or TNF-α group, ^#^*P* < 0.05 vs. TNF-α + AR or TNF-α + AR + DMSO group, ^$^*P* < 0.05 vs. TNF-α + AR + EETs group)

As shown in Fig. 12A and B, the expression of PI3K and p-AKT were significantly decreased both in vivo and in vitro experimental models. CYP2J2 overexpression and exogenous EETs significantly promoted the activation of PI3K/AKT pathway in vivo and in vitro.

## Discussion

In this study, we successfully established a rat model of PAH with LIRI and demonstrated that CYP2J2 overexpression and EETs could reduce inflammation, oxidative stress and apoptosis caused by PAH with LIRI, and also reduce pulmonary artery pressure and improve vascular remodeling in rats with PAH with LIRI.

Inflammatory response, oxidative stress and apoptosis play an important role during LIRI. Although LIRI could be reduced by anti-inflammatory, antioxidant and anti-apoptotic action [21], the development and treatment of LIRI are also affected by its comorbidities, especially when combined with PAH. PAH is characterized by pulmonary vasoconstriction and pulmonary vascular remodeling, narrowing and occlusion of small pulmonary arteries, and increased vascular resistance [22], which affect the reperfusion and aggravate the inflammatory response and lung injury. The endothelial cell dysfunction and in situ thrombosis caused by PAH increase thrombosis during lung ischemia [10, 23], which also reduces reperfusion and aggravates the inflammatory response and lung injury. Therefore, it is imperative to improve endothelial cell injury and vascular remodeling in PAH when treating LIRI in combination with PAH. In this study, we demonstrated that CYP2J2 overexpression and EETs could alleviate LIRI, when combined with PAH, through anti-inflammation, anti-oxidative stress and anti-apoptosis while improving PAH vascular remodeling.

In the inflammatory cascade caused by lung ischemia and reperfusion and subsequent activation of inflammatory cells, the activation of alveolar macrophages releases many inflammatory mediators which damage the vascular endothelium and alveolar epithelium. In the present study, we tested IL-1β and IL-6, which are directly involved in the initiation of lung injury by inducing early inflammatory responses, releasing toxic products and increasing lung vascular permeability as pro-inflammatory factors [24, 25]. Moreover, TNF-α stimulates the activation and aggregation of neutrophils [26], which also plays an essential role in acute lung injury. However, IL-10 is an anti-inflammatory factor that inhibits the inflammatory response of LIRI [27]. In this study, CYP2J2 overexpression and EETs significantly reduced the levels of IL-1β, IL-6 and increased the level of IL-10 while improving PAH with LIRI in vivo and in vitro. Therefore, CYP2J2 and EETs can protect against PAH with LIRI via anti-inflammatory effects. In addition, CYP2J2 overexpression and EETs in the study increased PPARγ levels and inhibited the phosphorylation of NF-κB in vivo and in vitro, suggesting that the anti-inflammatory effects may be related to the activation of PPARγ. PPARγ is considered to be the "gatekeeper" of extracellular matrix and vascular cell homeostasis, which helps maintain endothelial cell homeostasis and suppresses the inflammatory responses [28]. Our previous study showed that CYP2J2 and EETs could activate PPARγ and inhibit the downstream NF-κB activation, thereby regulating pro-inflammatory factors and cell adhesion molecules, suppressing inflammatory responses and inhibiting inflammatory cell adhesion to the vessel wall, but this protective effect was inhibited by GW9662 [15], a selective inhibitor of PPARγ. Furthermore, PPARγ can reduce PAH by decreasing inflammatory factor levels, inhibiting apoptosis and alleviating oxidative stress, and reducing vascular endothelial cell injury and vascular remodeling [29]. These data suggest that CYP2J2 overexpression and EETs may exert anti-inflammatory effects via PPARγ activation to attenuate PAH with LIRI.

The development of LIRI is also affected by oxidative stress and apoptosis. In the present study, we found that PAH with lung ischemia–reperfusion leads to ROS synthesis and further triggers mitochondria-associated events and apoptosis. ROS plays an important role in oxidative stress injury by causing structural damage to cells through protein inactivation, lipid peroxidation, and DNA damage [30]. In LIRI, the lack of oxygen supply during ischemia terminates ATP synthesis. Meanwhile, rapid ATP depletion leads to ATP-dependent ion pump dysfunction, decreased mitochondrial membrane potential, increased ROS synthesis, and triggered apoptosis [31, 32]. In lung ischemia, rapid ATP depletion leads to inactivation of ATP-sensitive potassium channels and free entry of sodium, calcium and water into the cell, causing endothelial cell membrane depolarization and abnormal endothelial cell function, accompanied by NADPH oxidase activation and consequently increased ROS synthesis and apoptosis. However, CYP2J2-EETs attenuated apoptosis caused by PAH with LIRI in this study. Simultaneously, we also found that CYP2J2-EETs increased the content of PI3K and phosphorylated AKT. The PI3K/AKT pathway is an important anti-apoptotic pathway. Activated AKT activates or inhibits several downstream apoptosis-related protein families (e.g., Bcl-2 family, BAX, etc.), thereby inhibiting apoptosis [33]. Our previous study showed that CYP2J2 and EETs could activate PI3K/AKT signal pathway and inhibit apoptosis in LIRI, but this protective effect was inhibited by LY294002 [14], a selective inhibitor of PI3K [34]. Feng et al. also showed that CYP2J2 and EETs could inhibited apoptosis in PAH via the activation of PI3K/AKT signal pathway in vivo and in vitro [17]. The above results suggest that CYP2J2 overexpression and EETs can activate PI3K/AKT signaling pathway, attenuate apoptosis in pulmonary artery endothelial cells, and protect against PAH with LIRI.

In addition, EETs, a sort of endothelium-derived hyperpolarizing factor (eEDHF), can maintain normal endothelial cell and vascular function and can relax vascular smooth muscle cells by activating Ca^2+^-sensitive K^+^ channels [35]. However, Strielkov et al. demonstrated that EETs relaxed pulmonary arteries in normoxia but constricted in anoxia [36]. In this study, CYP2J2 decreased the pulmonary artery pressure of the rat with PAH in combined with LIRI, which might be due to the vasoprotective effects of CYP2J2 and EETs on pulmonary arteries before IR, including relaxing pulmonary arteries and decreasing vascular remodeling. Furthermore, CYP2J2 and EETs can reduce the upregulation of cytokine-induced adhesion molecules, inhibit inflammatory cell adhesion to the vascular wall and suppress the migration of rat aortic smooth muscle cells [17]. In the present study, CYP2J2 gene transfection also effectively improved pulmonary artery pressure in rats with PAH combined with LIRI, and exogenous EETs improved endothelial cell injury treated with TNF-α and anoxia reoxygenation through the sequence. These results suggested that CYP2J2 overexpression and EETs could inhibit pulmonary vascular endothelial cell injury and reduce pulmonary hypertension.

Our study also has some limitations. Firstly, it is currently believed that LIRI has multiple pathogenic mechanisms, such as microvascular dysfunction, platelet activation, intracellular calcium overload, etc. Whether CYP2J2 and EETs act through other pathways needs further study. Secondly, the effects and mechanisms by which CYP2J2 and EETs act may vary depending on the concentration, but the control of CYP2J2 protein concentration could hardly be achieved by CYP2J2 gene transfection in this study, which also needs further study. In addition, the long-term effects of CYP2J2 and EETs in PAH with LIRI also need to be further investigated. Finally, the development of both PAH and LIRI is complicated. When PAH combined with LIRI, there is a possible interaction between them, still, their roles and mechanisms have not been fully clarified, and further studies are needed to clarify this interrelationship.

## Conclusions

CYP2J2 overexpression and exogenous EETs reduced PAH with LIRI through anti-inflammation, anti-oxidative stress, and anti-apoptosis in vivo and in vitro. The anti-inflammatory effects may be mediated by activation of PPARγ and the anti-apoptotic effects may be mediated by the PI3K/AKT pathway. CYP2J2 overexpression and EETs administration can be a new strategy for the prevention and treatment of PAH with LIRI.

## Data Availability

The datasets used and/or analysed during the current study are available from the corresponding author on reasonable request.
